# Impaired cardiac growth and function in children and adolescents after heart transplantation assessed by cardiac magnetic resonance

**DOI:** 10.1186/1532-429X-18-S1-O116

**Published:** 2016-01-27

**Authors:** Heiner Latus, Pauline Hachmann, Inga Voges, Samir Sarikouch, Brigitte Peters, Nona Mazhari, Kachina Behnke-Hall, Josef Thul, Hakan Akintuerk, Juergen Bauer, Christian Apitz, Dietmar Schranz

**Affiliations:** 1 University Children's Hospital Giessen, Germany, Pediatric Heart Centre, Giessen, Germany; 2 grid.10423.340000000095299877Department of Thoracic and Cardiovascular Surgery, Hannover Medical University, Hannover, Germany; 3 grid.5807.a0000000110184307Institute for Biometry and Medical Informatics, University of Magdeburg, Magdeburg, Germany

## Background

Cardiac growth after heart transplantation (HTx) in children has been reported to be normal in the long-term follow-up. The aim of our study was to evaluate ventricular volumes, mass and function in a cohort of pediatric HTx patients using cardiac magnetic resonance (CMR).

## Methods

Seventy-five pediatric HTx patients (mean age 14.0 ± 4.2 years, 26 females) were assessed by CMR 11.2 ± 5.4 years after HTx (time interval between HTx and CMR at least 1 year). Right (RV) and left ventricular (LV) volumes and LV mass were derived from short-axis cine images. The results were compared with a healthy reference population of 79 patients (mean age 13.7 ± 3.7 years, 29 females, p = 0.30) from the German Competence Network for Congenital Heart Defects. LV strain measurements were performed using CMR feature tracking (FT) software (TomTec, Germany) and were compared with a group of 46 healthy controls (mean age 13.3 ± 3.5 years, 21 females).

## Results

Both LV and RV enddiastolic and -systolic volumes were significantly smaller in the HTx group compared to healthy controls while LV mass was significantly higher in HTx patients. RV and LV ejection fraction were both reduced in HTx patients. While LV longitudinal (LS) and radial strain (RS) were significantly reduced, LV circumferential strain (CS) was preserved in the HTx group. LV-CS and LV-RS correlated with LV enddiastolic volume (r = -0.24, p = 0.04 and r = 0.25, p = 0.03). LV mass was inversely related to diastolic LV function assessed by early diastolic strain rate (r = -0.27, p = 0.02).

## Conclusions

Compared to healthy references, children and adolescents after HTx show impaired cardiac growth, increased myocardial mass and reduced global systolic pump function. Smaller LV dimensions are associated with impaired systolic strain while higher LV myocardial mass is related to diastolic function.

